# Characterization of photosynthetic *Bradyrhizobium* sp. strain SSBR45 isolated from the root nodules of *Aeschynomene indica*

**DOI:** 10.1080/15592324.2023.2184907

**Published:** 2023-03-06

**Authors:** Shingo Hata, Serina Kojima, Risa Tsuda, Nanami Kawajiri, Hiroshi Kouchi, Takamasa Suzuki, Kazuma Uesaka, Aiko Tanaka

**Affiliations:** a Faculty of Agriculture, Ryukoku University, Otsu, Japan; b Division of Arts and Sciences, International Christian University, Mitaka, Japan; c College of Bioscience and Biotechnology, Chubu University, Kasugai, Japan; d Center for Gene Research, Nagoya University, Nagoya, Japan; e Graduate School of Bioagricultural Sciences, Nagoya University, Nagoya, Japan

**Keywords:** *Aeschynomene indica*, *Bradyrhizobium*, fluorescence, genome, root nodule

## Abstract

We isolated a novel strain of *Bradyrhizobium* sp., SSBR45, from the nodulated roots of *Aeschynomene indica* and labeled it with *Discosoma* sp. red fluorescent protein (dsRED) or enhanced green fluorescent protein (eGFP) and determined its draft genomic sequence. The labeled SSBR45 stimulated the growth of *A. indica* markedly on a nitrogen-free medium, as observed by visualizing the fluorescent root nodules. The nodulated roots also exhibited high acetylene reduction activities. The SSBR45 genome included genes involved in nitrogen fixation, photosynthesis, and type IV secretion system; however, it did not consist of canonical *nodABC* genes and type III secretion system genes. SSBR45, a novel species of the genus *Bradyrhizobium*, consisted of an average nucleotide identity and average amino acid identity of 87% and 90%, respectively, with the closest strain *B. oligotrophicum* S58.

Most leguminous plants develop root nodules with nitrogen-fixing endosymbionts, called rhizobia; therefore, they can grow well on nitrogen-deficient soil. Legumes are presumed to have acquired this ability 60–70 million years ago.^1^, ^2^ In the initial stage of evolution, rhizobia are presumed to have colonized the leguminous roots through intercellular space,^2–4^ independent of the nodulation (Nod) factors.^4,5^ Both leguminous plants and rhizobial bacteria have co-evolved via an efficient process in which rhizobia enter through the infection threads in a Nod factor-dependent manner.^3^ *Aeschynomene indica* possesses the Nod factor-independent primitive root (and stem) nodule formation.^4^ Thus, the characterization of *A. indica* plants and endosymbionts of the family *Bradyrhizobiaceae* is essential to clarify the evolutional history of leguminous nodule formation and to apply the nitrogen-fixing ability to non-leguminous crops in the future. We isolated a new strain of *Bradyrhizobium* sp. from the nodulated roots of *A. indica* and named it SSBR45. This paper reports the draft genomic sequence and several properties of root nodules formed by SSBR45.

Fragments of bacterial 16S rRNA genes were amplified using a Bacterial 16S rDNA PCR Kit Fast 800 (Takara, Otsu, Japan). After determination of their sequences, phylogenetic trees were constructed using MEGA11 software.^6^ *pBjGroEL4::dsRED* was constructed from a mini-Tn5 plasmid^7^ as described previously.^8,9^ It contained the Shine-Dalgarno ribosome-binding site (5’-AGGAG-3’) between the promoter and fluorescent reporter gene and *trpA* terminator sequence (5’-AGCCCGCCTAATGAGCGGCTTTTTTTT-3’) downstream of the reporter gene. *pBjGroEL4::eGFP* was constructed by replacing *dsRED* with *eGFP*,^10^ and the sequence from the beginning of the *BjGroEL4* promoter to the end of the *eGFP* coding region was deposited to the DNA Data Bank of Japan (DDBJ)/European Molecular Biology Laboratory (EMBL)/GenBank under accession number LC741218. The plasmids were introduced into *Escherichia coli* S17-1 λ*pir*, and transferred to SSBR45 by biparental conjugation, the mini-transposons being inserted into the chromosome. The colonies of fluorescence-labeled SSBR45 were recovered on an HEPES–MES (HM) agar plate^11^ containing streptomycin, spectinomycin, and phosphomycin (50 µg/mL each). Phosphomycin was added for counter selection of *E. coli*. Hereafter, the dsRED- and eGFP-labeled strains were named as SSBR45R and SSBR45G, respectively. The bacterial colonies were observed with a SZ61 stereomicroscope (Olympus, Tokyo, Japan) equipped with a BT-S&IB fluorescence-detection unit (BioTools, Takasaki, Japan). The dsRED was detected through a red filter using an excitation LED light (530 nm), and eGFP was detected through an orange filter with excitation light (485 nm). The pictures were captured with a TrueChrome II plus camera (BioTools). Surface-sterilized *A. indica* seeds were germinated on sterile filter papers in a cultivation box, grown for 2 days in the dark, and transplanted into a sterile Leonard jar assembly with double polycarbonate boxes^12^ filled with vermiculite and 0.5 × Broughton & Dilworth (B&D) medium.^13^ The SSBR45 cells were cultured for 4 days in HM medium,^11^ and approximately 2 × 10^6^ cells/plant were inoculated just after transplantation. The *A. indica* plants were grown in a green room at 27°C in a 16 h light (130 µmol s^−1^ m^−2^)/8 h dark cycle. The acetylene reduction activity of each plant root was determined as described previously for *Nostoc* colonies.^14^ The outward appearance of fluorescent nodules was observed with a SZ61 stereomicroscope (Olympus) as described above. The nodulated roots of *A. indica* were embedded in 5% agar, and sections of 60 µm thickness were prepared with a DTK-1000 N microslicer (Dosaka EM, Kyoto, Japan) for laser scanning confocal microscopy. A TCS SP8 DMi8 microscope equipped with an HCX PL APO CS2 10x/0.40 objective lens (Leica, Heidelberg, Germany) was used for single-photon microscopy with simultaneous excitation by 552 nm and 488 nm lasers, and concurrent detection of dsRED and eGFP was performed as reported previously.^15^ The genomic analyses of SSBR45R and SSBR45G were performed as described previously for *Ralstonia* sp.^16^

Besides nitrogen-fixing rhizobia, many other co-existing bacteria are present in the leguminous root nodules.^17,18^ We isolated the bacterial strains from nodulated roots of local *A. indica*, as reported previously,^16^ and identified them based on the 16S rRNA gene sequences. The first and second strains were *Ralstonia* sp. SET104^16^ and *Pantoea* sp. (unpublished data), respectively. The third one was *Bradyrhizobium* sp., SSBR45, with pale-pink colonies (Supplementary Figure S1a and c). A gene fragment of *nifH* that encodes a nitrogenase subunit was amplified by colony PCR of SSBR45, using PolF/PolR primers,^19^ unlike the other two strains (data not shown). Thus, SSBR45 was expected to perform nitrogen fixation. A detailed phylogenetic tree showed that SSBR45 is a member of photosynthetic *Bradyrhizobium*, like ORS278,^5^ BTAi1,^5^ ORS285,^4^ and *B. oligotrophicum* S58^8^ (Figure 1). As the first step to characterize SSBR45, we labeled it with dsRED or eGFP (Supplementary Figure S1b and d). Both SSBR45R and SSBR45G markedly promoted the growth of *A. indica* on a nitrogen-free medium (Figure 2a). The mean dry weights of non-inoculated and inoculated seedlings were 90 mg/plant and 273 mg/plant, respectively. Non-fluorescent SSBR45 showed similar growth promotion to SSBR45R and SSBR45G, suggesting that the fluorescent markers did not affect the nodule-forming and nitrogen-fixing activities of SSBR45. The root nodules were formed at the bases of lateral roots as described previously,^4^ while stem nodules were rarely formed under our conditions. The acetylene reduction activity of nodulated roots inoculated with either SSBR45R or SSBR45G was around 215 nmol/plant/10 min, whereas that of non-inoculated control roots was not detectable. The fluorescence inside of the nodules was occasionally observed, probably because the epidermis and cortex of *A. indica* nodules are rather thin (Figure 2b,c). The inspection of the inside of nodules formed by simultaneous inoculation of SSBR45R and SSBR45G showed that the central infected zone consists of the infected cells only, and that most of them were derived from a single or very few bacteria-infected founder cortical cells (Supplementary Figure S2a and b). In addition, the fused nodules of two closely emerging ones were found, although the frequency was not high (Supplementary Figure S2c). These results confirmed a previous report.^4^

**Figure 1.**
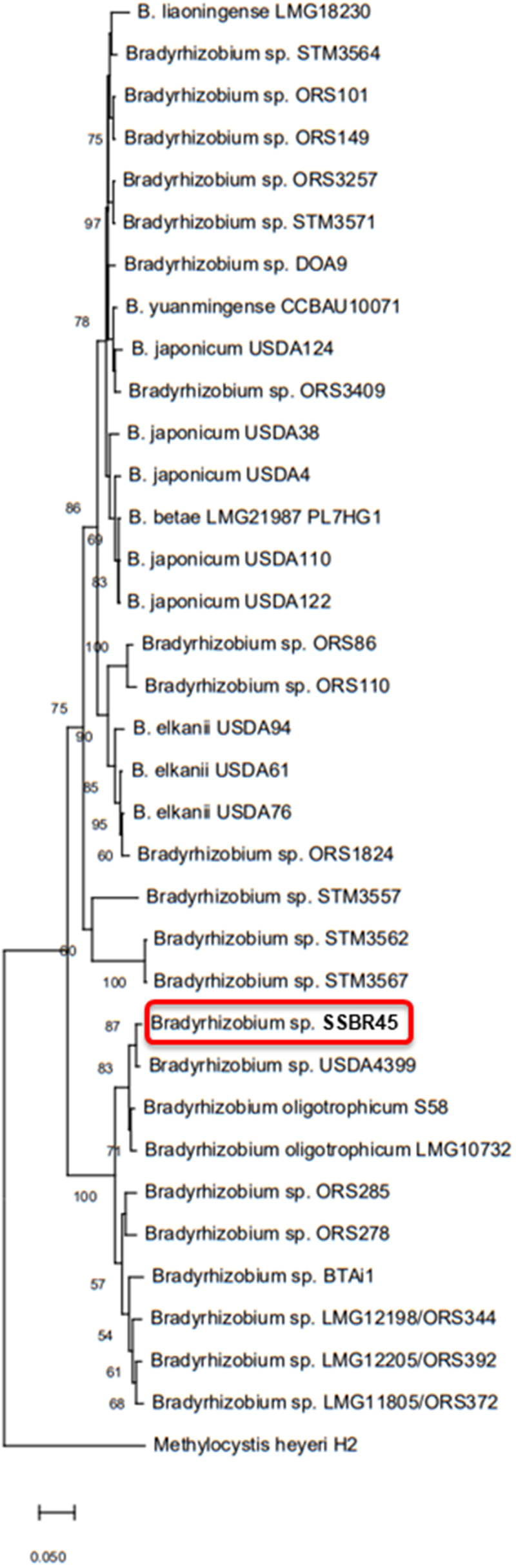
A phylogenetic tree of representative members of the genus *Bradyrhizobium* based on the 16S rRNA gene sequences. The bootstrap values were expressed as the percentage of 1,000 replications. The evolutionary distances were computed using the Kimura two-parameter method.^20^ The bar represents 50 estimated substitutions per 1000-nucleotide positions.

**Figure 2.**
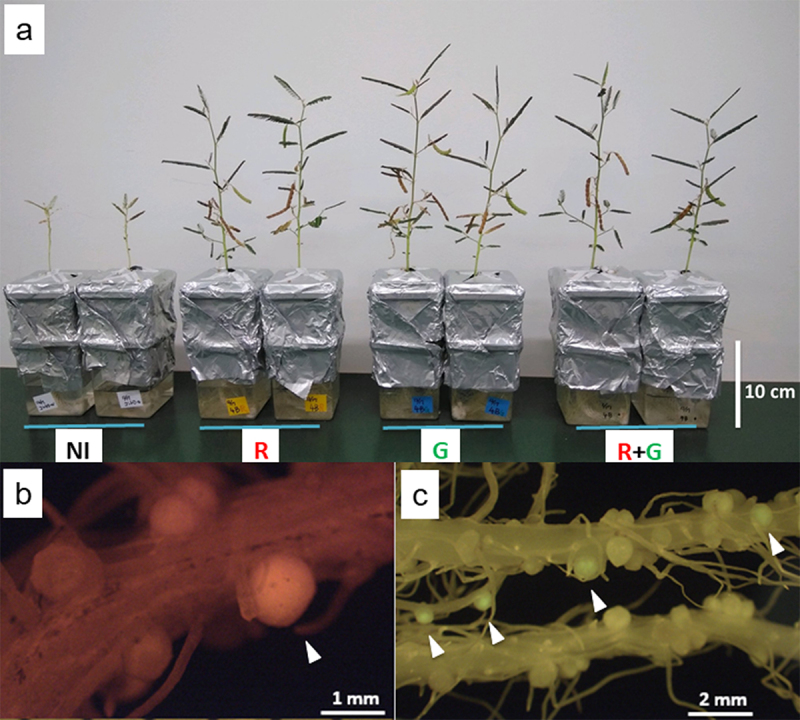
The growth of *A. indica* and root nodules formed by SSBR45. (a) The plants on vermiculite were grown in a nitrogen-free medium. NI, non-inoculated control; R, seedlings inoculated with SSBR45R; G, seedlings inoculated with SSBR45G; and R + G, seedlings inoculated with an equivalent mixture of SSBR45R and SSBR45G. The mean dry weights of non-inoculated and inoculated seedlings were 90 mg/plant and 273 mg/plant, respectively. They were grown for seven weeks after the inoculation. (b) A fluorescing nodule and dark nodules were formed by SSBR45R. (c) The fluorescing nodules and dark nodules on the roots inoculated with SSBR45G (upper root) and non-fluorescent SSBR45 (lower root). Arrowheads indicate the fluorescing nodules. The pictures of (b) and (c) were taken four weeks post-inoculation. The causative structural difference between fluorescing and dark nodules is still unknown.

The draft genomic sequences of SSBR45R and SSBR45G were determined by the method described previously,^16^ and deposited in the DDBJ/EMBL/GenBank under accession numbers BSCU01000001-BSCU01000084 and BSCT01000001-BSCT01000083, respectively. The SSBR45R sequence consisted of 8,251,682 bases with a GC content of 65.6% and 7,447 putative coding sequences, while the SSBR45G sequence consisted of 8,252,498 bases with a GC content of 65.6% and 7,448 putative coding sequences. A single copy each of *dsRED* gene and *eGFP* gene was found in the SSBR45R and SSBR45G genomes, respectively. The nitrogen fixation and photosynthetic gene clusters of SSBR45 were very similar to *B. oligotrophicum* S58 and *Bradyrhizobium* sp. ORS278, although ORS278 has an extra transposase gene in each cluster (Figure 3a,b). The location of these insertion sequences near to important genes involved in symbiosis was noticeable. If some genes were knocked out, it would be disadvantageous for the *Bradyrhizobium* strains in regard to survival. Notably, the SSBR45 genome did not consist of canonical *nodABC* genes, essential for synthesizing the Nod factor. Also, the type III secretion system gene that affects the specificity of symbiotic partners for nodule formation^22^ was not found in its genome, like ORS278, BTAi1, and *B. oligotrophicum* S58. In contrast, type IV secretion system genes were found in the SSBR45 genome, the kind and number being equal to those found in the *B. oligotrophicum* S58 genome (Supplementary Table S1). The type IV system was recently reported to be involved in the nodule-forming activities of *Sinorhizobium* and *Mesorhizobium*;^23,24^ however, its role in photosynthetic *Bradyrhizobium* strains, such as SSBR45 and S58, remains to be elucidated. The genomic sequences of USDA4399 and LMG10732 have not been reported; thus, the strain closest to *B. oligotrophicum* S58 was SSBR45 (Figure 1). The average nucleotide identity (ANI) and average amino acid identity (AAI)^25–27^ were 87% and 90%, respectively, in relation to the closest strain (Supplementary Tables S2 and S3). Genomes with an ANI of >95% and/or an AAI of >95% are considered to have originated from the same species.^25–27^ Therefore, we concluded that SSBR45 is a novel species of the photosynthetic *Bradyrhizobium* genus. Since *Bradyrhizobium* is estimated to contain as many as 800 species,^28^ extensive efforts to discover new species in the genus must be continued. Recently, important progress has been made in the research into plant signaling for the unique process that is independent of Nod factors and infection threads. Using *Aeschynomene evenia* and several other *Aeschynomene* species, Quilbé et al. revealed that a cysteine-rich receptor-like kinase gene and most common symbiotic signaling genes were required for the process.^29^ They also reported that genes for Nod factor receptors and exopolysaccharide receptors were missing or not working in these host plants.^29^ However, the bacterial determinant of the Nod-independent nodule formation of some *Aeschynomene* species, which may be the ligand of the cysteine-rich receptor-like kinase, has not yet been elucidated. Therefore, it will be interesting to seek a common working gene among SSBR45, ORS278, BTAi1, and S58. Another possibility would be bacterial forward genetics; for example, via transposon tagging of the SSBR45 genome. If the SSBR45 mutants that do not perform intercellular infection into *Aeschynomene* species are discovered, the mutants will contribute greatly to the clarification of the Nod-independent nodule formation mechanism of some *Aeschynomene* species.

**Figure 3.**
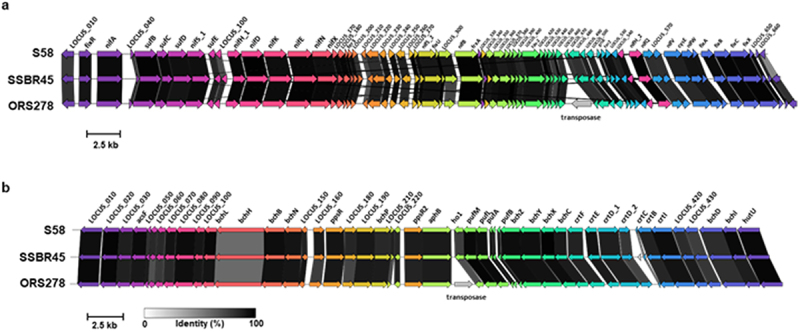
The comparison of (a) nitrogen fixation and (b) photosynthetic gene clusters of SSBR45, S58, and ORS278. The analysis was performed using the “clinker” program (https://pypi.org/project/clinker).^21^

## Supplementary Material

Supplemental Material
