# Supplementary figures and images for: Characterization of photosynthetic Bradyrhizobium sp. strain SSBR45 isolated from the root nodules of Aeschynomene indica
Source: Plant Signal Behav. 2023 Mar 6;18(1):2184907. doi: 10.1080/15592324.2023.2184907 (PMC10012927; doi:10.1080/15592324.2023.2184907)

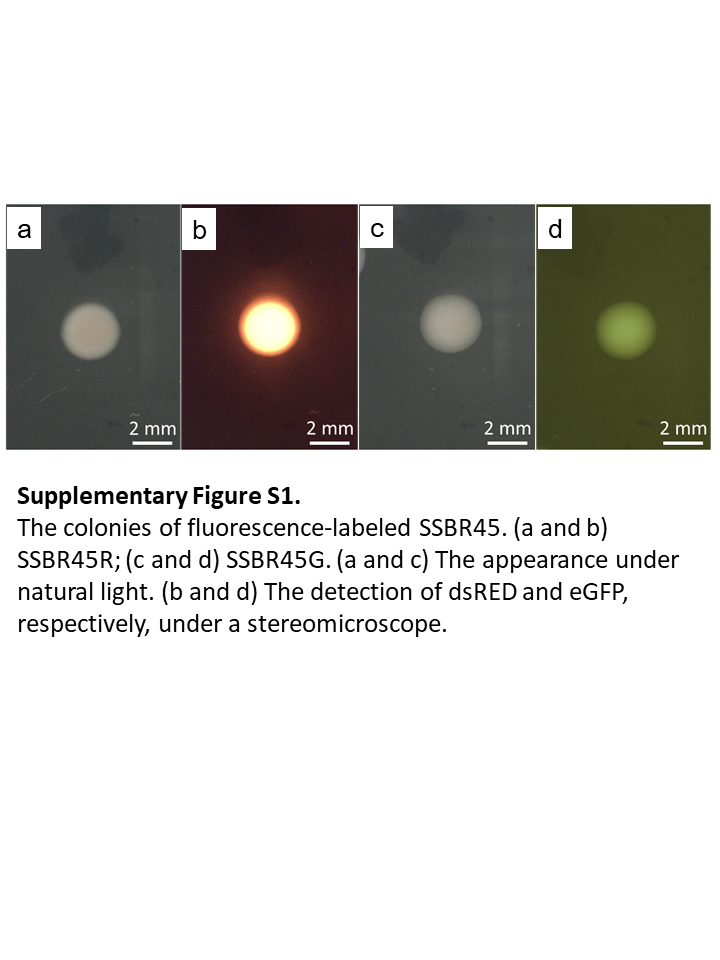

Supplement: Supplemental Material [file KPSB_A_2184907_SM5660.zip › KPSB_2022_0231R1_SupplFigS1.TIF]

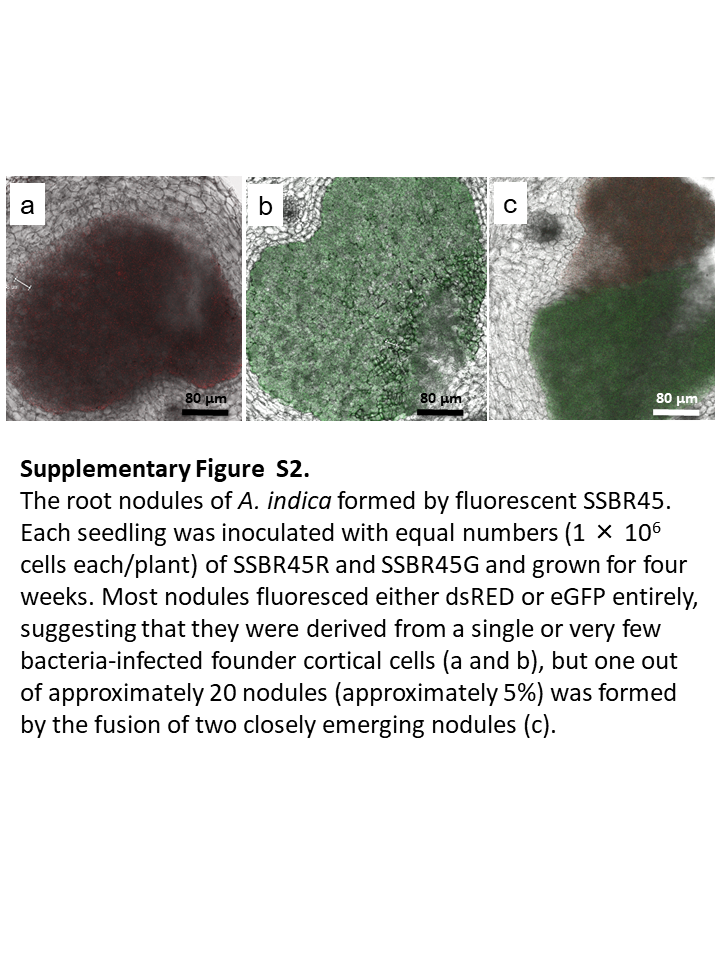

Supplement: Supplemental Material [file KPSB_A_2184907_SM5660.zip › KPSB_2022_0231R1_SupplFigS2.TIF]

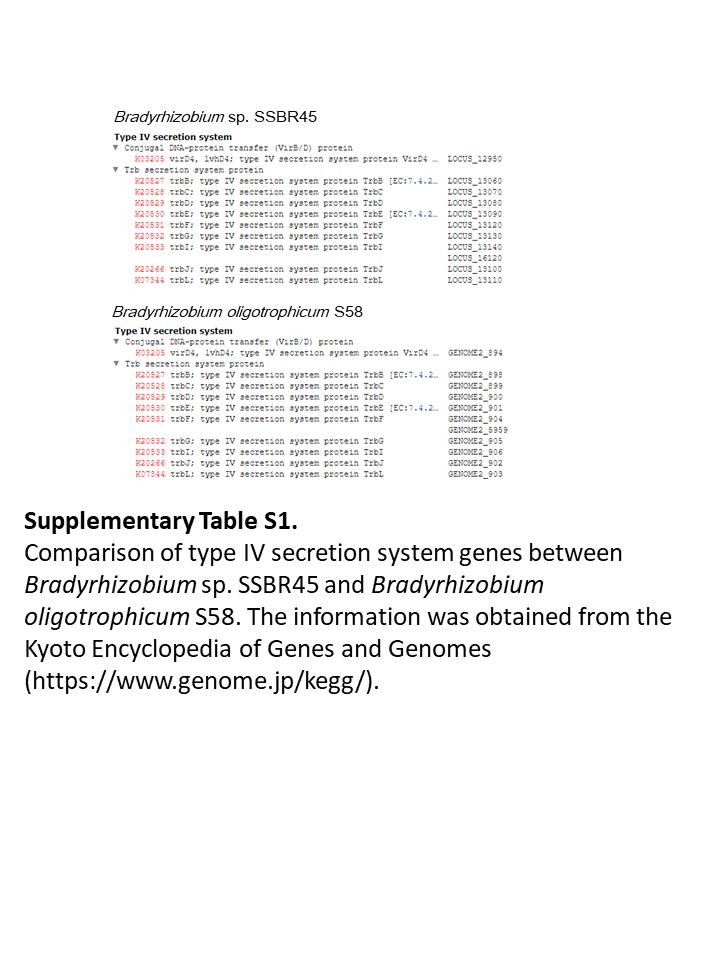

Supplement: Supplemental Material [file KPSB_A_2184907_SM5660.zip › KPSB_2022_0231R1_SupplTabS1.TIF]

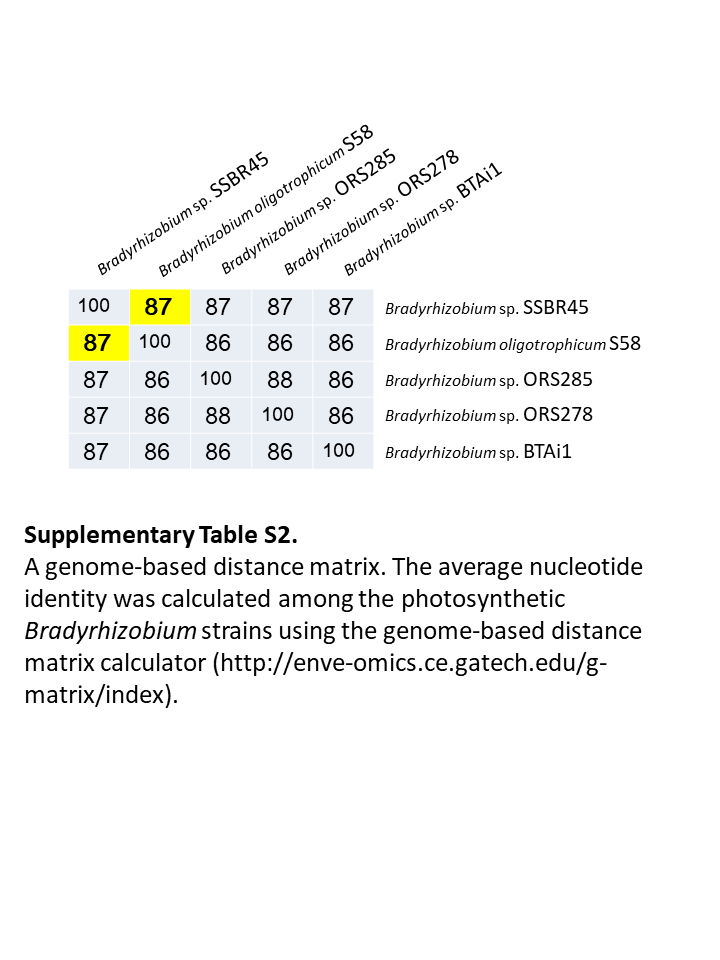

Supplement: Supplemental Material [file KPSB_A_2184907_SM5660.zip › KPSB_2022_0231R1_SupplTabS2.TIF]

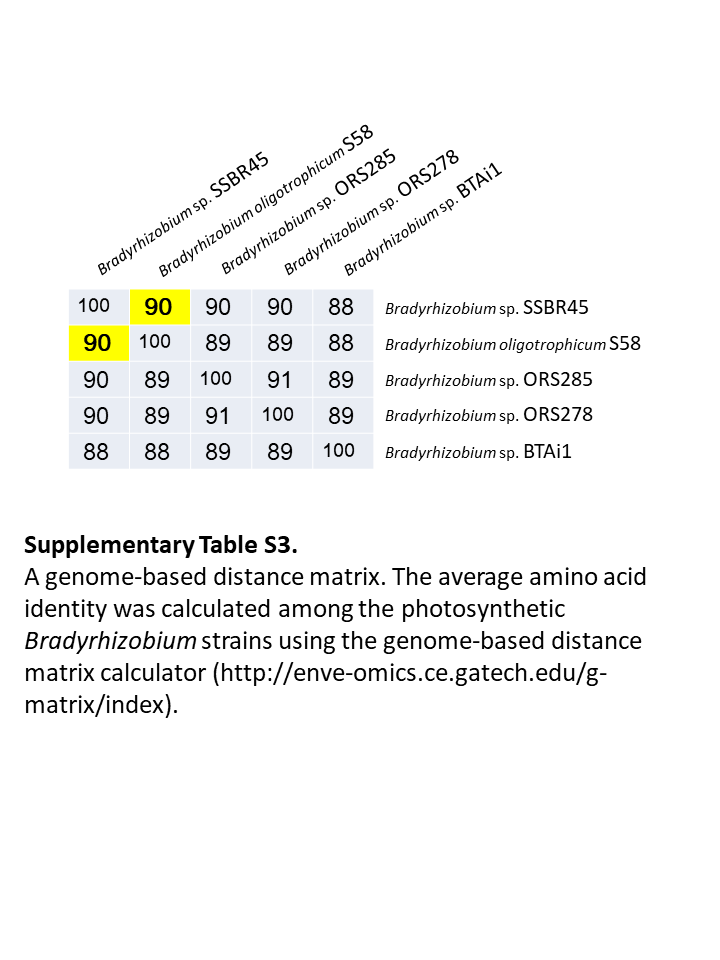

Supplement: Supplemental Material [file KPSB_A_2184907_SM5660.zip › KPSB_2022_0231R1_SupplTabS3.TIF]
